# From strains to ecosystems: biocrust microbiomes as a new paradigm for dryland agriculture

**DOI:** 10.1093/ismejo/wrag174

**Published:** 2026-07-02

**Authors:** Corey Nelson, Fernando T Maestre

**Affiliations:** Biological and Environmental Science and Engineering Division, King Abdullah University of Science and Technology, Thuwal 23955-6900, Kingdom of Saudi Arabia; Biological and Environmental Science and Engineering Division, King Abdullah University of Science and Technology, Thuwal 23955-6900, Kingdom of Saudi Arabia

**Keywords:** biocrust, cyanosphere, drylands, marginal lands, PGP, regenerative agriculture, bioinoculants, applied microbiology

## Introduction

Drylands cover over 40% of Earth’s terrestrial surface, strongly influence global carbon and nitrogen cycles, and support more than 2 billion people through crop and livestock production [1]. As climate change intensifies water scarcity, soil degradation, and heat stress across these regions, the need for resilient nature-based strategies to sustain soil function and agriculture under chronic environmental stress is becoming increasingly urgent. In these regions, declining soil function and increasing dependence on external inputs threaten not only productivity, but also the stability of food systems and livelihoods that already operate close to environmental limits [2]. Many of the soil functions most threatened by climate change in drylands are fundamentally shaped by microbial activity; thus, microbial strategies should become a central target for adaptation in these regions [3]. Here, we argue that a major limitation of dryland bioinoculation strategies is not the lack of beneficial microbial traits, but the reliance on strain-based designs poorly matched to the ecological characteristics of dryland soils. We propose biocrust microbiomes as a natural template for a different approach: locally adapted, multifunctional microbial ecosystems shaped by natural selection for persistence, cooperation, and soil-health building within circular, regenerative dryland agricultural systems. More broadly, we argue that dryland agriculture provides a test case for a wider question in applied microbial ecology: whether durable microbiome-based interventions should be designed around ecological organization and community assembly rather than around portable traits in isolated strains.

Among the microbial strategies proposed for improving dryland agriculture, bioinoculation has attracted particular attention for its potential to renew nitrogen availability, mobilize phosphorus, stabilize organic carbon, and enhance crop yields and drought tolerance. Yet most commercial bioinoculants have been developed for humid, high-input cropping systems and are poorly suited to dryland agriculture, where soils are characterized by extreme temperature fluctuations, low organic matter, high pH, and low moisture retention [4]. Unsurprisingly, many of these inoculants fail to establish or maintain beneficial functions under such stress [5]. The key challenge, therefore, is not simply to identify microbes with desirable traits, but to find microbial systems capable of persisting and functioning in dryland soils. In this context, biological soil crusts (biocrusts) emerge as a compelling model of the kind of microbial system likely to succeed in dryland agriculture: communities shaped by strong environmental selection to endure arid conditions and simultaneously enhance soil stability, nutrient cycling, and plant performance [6, 7].

### Biocrusts naturally promote soil health in drylands

Biocrusts are photoautotroph-driven communities that occupy the upper millimeters of arid and semi-arid soils, where they persist under chronic desiccation, intense solar radiation, nutrient scarcity, and extreme temperature fluctuations [8]. Composed of cyanobacteria, algae, lichens, bryophytes, and associated heterotrophic fungi, bacteria, archaea, and viruses, biocrusts provide a natural example of how microbial communities can remain both persistent and multifunctional under chronic dryland stress. Their resilience emerges from organized communities structured by metabolic exchange, cooperation, and protective traits such as UV-screening pigments, osmolytes, and extracellular polymers [8, 9], features especially relevant to agricultural soils exposed to the same stresses.

Biocrust resilience at the community level translates into multiple functions that directly shape soil health in dryland ecosystems. First, biocrusts act as soil biofertilizers: cyanobacteria and other phototrophic constituents supply carbon through photosynthetic fixation and exudation, whereas both phototrophic and heterotrophic microbes contribute fixed nitrogen and support nutrient cycling in otherwise resource-poor soils [10]. In parallel, nutrient-mobilizing members of biocrust microbiomes can improve access to poorly available micronutrients such as phosphorus, reinforcing their potential as naturally multifunctional biofertility systems [8, 9]. Second, they engineer the physical soil environment: exopolysaccharides produced by biocrust microbes reduce erosion by binding soil particles into stable aggregates and form cohesive surface biofilms that buffer rapid wet-dry cycles, slow evaporation, and enhance the retention of water and deposited nutrients [10]. Overall, biocrusts function as integrated microbial systems that create the biological, physical, and hydrological conditions necessary to preserve and rebuild soil fertility and stability in dryland ecosystems.

### Microbial cooperation drives resilience in biocrusts

**Figure 1:**
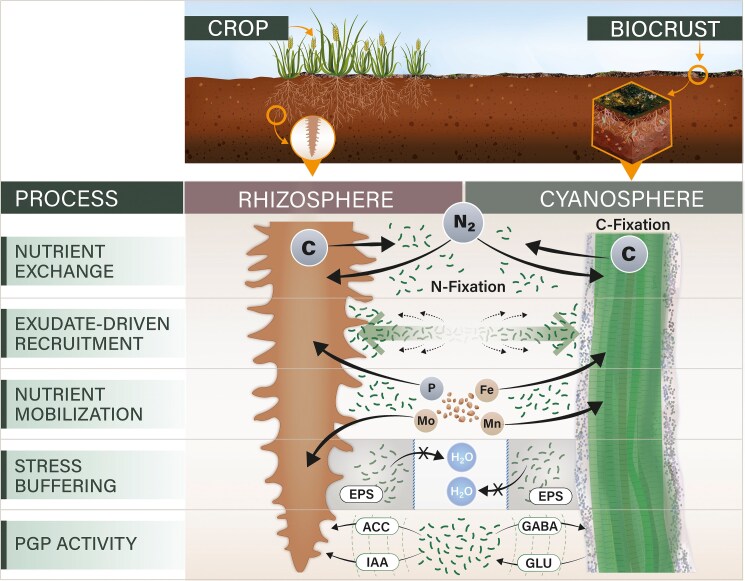
Conceptual parallels between the plant rhizosphere and the biocrust cyanosphere. In both systems, host-derived exudates recruit heterotrophic partners that contribute to nutrient exchange such as carbon (C) and nitrogen (N), nutrient mobilization (phosphorus and micronutrients), stress buffering through osmolytes and exopolysaccharides (EPS), and plant growth-promoting (PGP) activity (phytohormones and extracellular enzymes) to promote persistence under environmental stress. Figure abbreviations: Indole-3-acetic acid (IAA), ACC deaminase (ACC), glutamate (GLU), and gamma-aminobutyric acid (GABA).

The success of biocrusts in drylands depends on microbial interactions that structure their development and persistence. A key example is that biocrust-forming pioneer cyanobacteria such as *Microcoleus vaginatus* and other members of the *Coleofasciculaceae* maintain closely associated heterotrophic communities enriched in traits linked to nutrient acquisition and stress tolerance, collectively termed the cyanosphere (in analogy to the plant rhizosphere) [11]. In biocrusts, cyanobacteria form dense filament bundles that initially stabilize soils and create carbon-rich microsites; like roots, they exude organic acids, amino acids, and polysaccharides that recruit and maintain beneficial microbial partners [12, 13]. This analogy is mechanistically important because both systems are structured by exudate-driven recruitment of heterotrophs that support nutrient mobilization, stress buffering, and community persistence. As a result, both the cyanosphere and rhizosphere harbor distinct communities enriched in genera with plant growth-promoting traits, including nitrogen fixation, phosphate solubilization, and phytohormone production (Fig. 1) [14, 15]. This parallel also raises the possibility that biocrust-derived microbes may integrate well into crop-associated soils in drylands. Supporting this view, the diversity–function relationship in natural biocrusts shows that multi-species assemblages sustain greater soil stabilization and fertility than single taxa [16] and, translating this to application, biocrust-derived consortia can outperform isolated strains, with associated heterotrophs supporting cyanobacterial function under nutrient stress [15, 17]. In addition to these cooperative interactions, the constituent microorganisms themselves have evolved under chronic exposure to harsh environmental conditions, selecting for exceptional hardiness and prolonged viability in a dried state. This intrinsic stress tolerance may improve inoculant survival under field conditions and also make biocrust-derived communities attractive for long-term storage, transport, and establishment in arid regions. Together, this pairing of cooperative interactions and intrinsic stress tolerance is what a dryland inoculant would need to retain to survive and function in the field.

### From single strains to ecosystem-level inoculation in drylands

Early applied studies further suggest that biocrust-derived inoculation can improve soil microbial diversity, water retention, and soil carbon [10]. However, these results remain largely proof of concept and do not yet show whether introduced communities can establish sustainably or generate lasting gains in soil function under field conditions. Current bioinoculation strategies often rely on single strains or simplified mixtures selected for obvious traits such as nitrogen fixation or stress tolerance, but removed from their ecological context. Many biocrust restoration efforts using cultured cyanobacteria have struggled to establish once returned to field conditions, a pattern consistent with the limitations of strain-centric approaches and a central motivation for the ecosystem-level strategy we propose here [7, 16]. Within drylands, biocrust ecology points to a different design logic. In natural biocrusts, community composition and long-term stability depend on co-adapted interactions, functional redundancy, spatial organization, and network robustness among phototrophic and heterotrophic partners [11, 12]. Therefore, the relevant target of intervention should not be isolated traits or taxa, but the entire microbial ecosystem that sustains persistence and multifunctionality in dryland soils. The limitations of current methods are especially clear when inoculation efforts prioritize later-successional or functionally desirable taxa, particularly nitrogen-fixing cyanobacteria such as *Nostoc, Tolypothrix*, and *Scytonema*, without considering the assembly processes that allow these organisms to establish in the first place [10]. In natural biocrust succession, pioneer bundle-forming cyanobacteria stabilize the soil surface and modify moisture and nutrient conditions, enabling later-stage organisms, such as nitrogen-fixing cyanobacteria, to persist [9]. Without that ecological foundation, inoculation may generate only short-lived responses that require repeated application, effectively functioning more as an expensive biofertilizer than as a durable, self-sustaining soil-health intervention.

Large-scale cultivation remains a major obstacle to this ecosystem-level approach. Biocrust production has historically been constrained by cost, land demand, and water use, but recent proposals for “crustivoltaics,” the co-location of solar infrastructure and biocrust cultivation, suggest a plausible path forward, although their economic and logistical feasibility at field scale remains to be demonstrated [18]. In principle, biocrust biomass could be cultivated locally on non-arable land associated with solar or urban infrastructure, using non-potable water sources such as treated sewage effluent, and then transferred to nearby marginal agroecosystems as a living amendment. This model would address not only a production bottleneck, but also an ecological one. Because cultivation would occur near the site of application, biocrust-derived communities could be propagated under environmental conditions closer to those of deployment and with greater retention of locally adapted taxa. In turn, this may improve inoculant establishment and avoid both the need to harvest biocrust from intact landscapes and the ecological risk of introducing non-native communities, key sustainability concerns for any biocrust-based amendment.

Locally matched cultivation may be especially compelling in arid agricultural systems, where managed water inputs help overcome one of the main constraints on biocrust establishment. Routine crop irrigation already provides repeated wetting cycles, creating a managed window in which biocrust establishment and associated soil benefits could develop on agricultural rather than purely natural timescales. If established, these communities may enhance soil aggregation, increase water retention, promote nutrient accumulation, and foster more resilient soil microbiomes. Over time, they may also reduce dependence on irrigation, fertilizers, and repeated microbial inputs by rebuilding the biological infrastructure of the soil itself. Viewed this way, biocrust-derived inoculation becomes part of a circular and regenerative strategy in which underused land, lower-quality water, and renewable-energy infrastructure are repurposed to cultivate microbial ecosystems that restore soil function and reduce future external inputs.

Cultivated systems impose pressures distinct from those shaping biocrusts in undisturbed drylands: some threaten establishment, whereas others shape which community persists. The most immediate is physical disturbance. Tillage, traffic from heavy machinery even in nominally no-till systems, and livestock trampling are all known to fragment biocrusts in natural settings [8], and would likely impose repeated setbacks on establishment in agricultural fields. Yet those same disturbance regimes also act as filters on community composition, as do irrigation, fertilization, soil type, the resident soil microbiome (which can impose biotic resistance to incoming taxa), and crop species. Rather than only a barrier, this selective environment may reframe the goal of inoculation: the favored organisms could be taxa that are minor in natural biocrusts yet remain locally adapted and native, and better suited to agricultural conditions while still delivering moisture retention, surface stabilization, and nutrient inputs. The relevant target may therefore not be the natural community’s composition, but the configuration of its locally adapted members that performs best under agricultural selection. Whether biocrust-derived communities persist as more than expensive biofertilizers will then depend on whether these conditions enrich beneficial soil-building taxa or instead favor weedy phototrophs, an open question that field studies, not greenhouse analogues, must resolve.

### Research priorities for translating biocrust microbiomes into dryland agriculture

**Figure 2:**
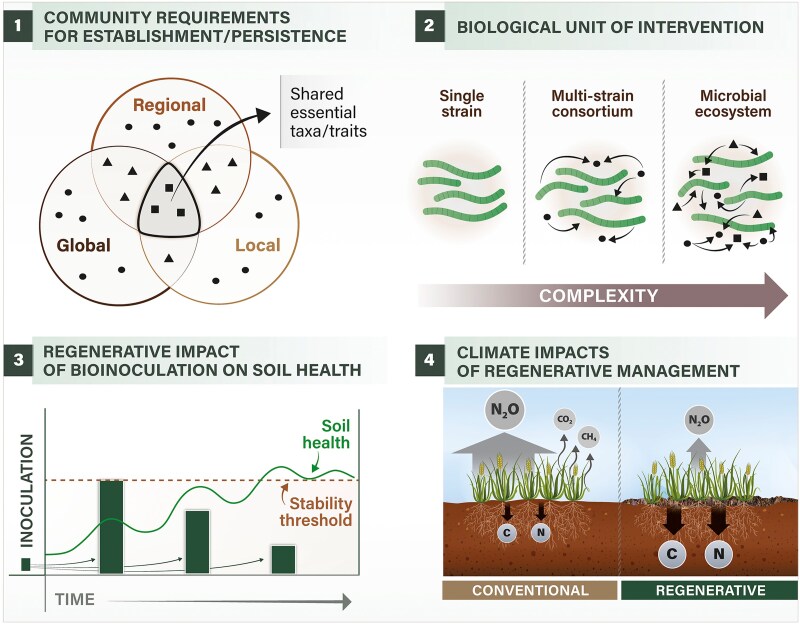
Four key questions that must be addressed to enable the effective use of biocrusts in agriculture. These include: (1) how microbial community composition and functional traits vary across global, regional, and local scales, and which of these are essential for biocrust growth and establishment in soils; (2) the degree of community organization and biological interaction required for biocrust-based bioinoculants to persist after introduction into soil; (3) whether biocrusts can improve soil health over time to a threshold at which regenerative effects become self-sustaining; and (4) how biocrusts influence soil carbon stabilization and greenhouse gas fluxes in agricultural systems managed under regenerative practices.

Despite decades of research on biocrust ecology, we still lack the molecular and ecological framework needed to determine whether these microbial systems can function as durable agricultural interventions. Existing work has described biocrust succession on bare soils, including which organisms colonize early, which appear later, and what ecosystem functions they support [8]. However, much of this literature remains largely observational, marker-gene-based, or focused on a limited set of traits in natural or restoration contexts. Metagenomic analyses of biocrusts and their cyanosphere microhabitats also remain limited, leaving unresolved the genomic basis of multifunctionality, long-term stability, and compatibility with crop-associated soils. The next phase of the field should therefore be organized around four key questions (Fig. 2), with the central test being whether biocrust-derived inoculants outperform conventional inoculants or amendments in persistence, multifunctionality, and durability under realistic dryland management. Although framed around dryland agriculture, these questions also ask whether ecological organization itself can serve as a transferable design principle for durable microbiome-based interventions.

#### Which taxa, functions, and interactions are essential for establishment and persistence in agricultural soils?

Although biocrust succession has been described in natural systems, it remains unclear which taxa, functions, and interactions are causally required for biocrust establishment, persistence, and recovery in agricultural soils exposed to irrigation, fertilization, and repeated disturbance. Addressing this gap will require moving beyond descriptive succession studies to experiments that identify which microbial groups act as pioneers, which later-stage taxa depend on them, and which interaction networks are necessary to assemble stable biocrust-derived communities under managed dryland conditions. Standardized sampling, sequencing, and analytical frameworks will be essential for this effort, and initial steps toward a systematic global evaluation of biocrust communities are already underway through efforts such as CrustNet (https://crustnet.org/). Such standardization should help distinguish broad global and regional patterns from local site-scale variability, and also make it possible to test how inoculant provenance influences establishment, including whether locally derived communities outperform taxa sourced from climatically distinct regions. Together, these comparative frameworks, combined with genome-resolved metagenomics, time-resolved community profiling, cultivation, and manipulative assembly experiments, could reveal the mechanisms that govern biocrust establishment and resilience across environmental gradients. Identifying those mechanisms would be a major milestone for applied microbial ecology and for determining whether biocrust-derived communities can deliver sustained soil-health benefits in agriculture.

#### What is the appropriate biological unit of dryland bioinoculation?

Even if key taxa and functions can be identified, the field still needs to determine the appropriate biological unit of dryland bioinoculation. Does success depend on selected strains, designed consortia, faithfully propagated whole communities, or a diverse, locally adapted source community shaped by agricultural selection itself? Most biocrust-based inoculation studies have focused on single strains, and only a limited number have attempted phototroph/heterotroph consortia [17], despite evidence that biocrust function in nature emerges from spatially organized, metabolically connected communities [12, 13]. Future work should therefore compare these units under the same agricultural conditions, evaluating both short- and long-term responses (weeks to years) on establishment, persistence, rebuilding of soil structure, nutrient cycling, and microbial stability. The most informative comparisons would ask not only whether greater initial diversity and organization improve outcomes, but whether a given management regime reproducibly selects the same persistent, soil-beneficial configuration from a broad source pool, and whether that regime can be tuned to steer which configuration emerges. If propagated communities consistently outperform isolates or simplified mixtures, it would identify the microbial ecosystem, not the individual strain, as the functional unit of dryland bioinoculation.

#### Do biocrust-derived communities generate cumulative and lasting improvements in soil health under agricultural management?

A central unresolved question is whether biocrust-derived communities can generate cumulative and lasting improvements in soil health rather than short-lived functional gains in agricultural systems. Addressing this will require tracking how inoculated communities influence soil aggregation, water retention, nutrient accessibility, microbial diversity, and crop performance across repeated growing cycles, and also testing whether irrigation, fertilization, surface disturbance, crop shading, and residue management alter the assembly and persistence of biocrust-derived communities in agricultural soils. It will also be important to determine whether these effects vary among crops that differ in rooting strategy, nutrient demand, and rhizosphere chemistry. More specifically, the field needs to test whether biocrust inoculation initiates self-reinforcing changes that persist over time and reduce dependence on repeated inputs. It will be essential to determine how many applications are required for biocrust establishment, how long functional benefits persist after inoculation, and whether there is a threshold beyond which only minimal further inputs are needed. In practice, success would mean that these communities become lasting components of the soil system, rebuilding structural stability, microbial connectivity, and nutrient-retention capacity.

#### What are the consequences for greenhouse-gas fluxes and carbon stabilization?

Biocrust-derived inoculants may influence not only soil health, but also the broader climate consequences of agricultural management. Because agriculture strongly alters carbon and nitrogen cycling, any intervention that changes how nutrient inputs are transformed, retained, or lost in dryland agroecosystems could have consequences beyond the field scale. For example, agricultural soils can lose substantial nitrogen through gaseous emissions under episodic wetting and fertilization [19]. Future work should therefore test how biocrust-derived communities influence nitrogen transformations, soil redox dynamics, and net fluxes of gases such as N_2_O, CO_2_, and CH_4_. Their effects on soil carbon stabilization, erosion, and dust suppression should likewise be quantified under realistic field conditions. The guiding question should be whether these communities can improve soil function locally and also shift dryland agroecosystems toward lower nitrogen losses, greater carbon retention, and more stable soil surfaces under climate stress. If so, biocrust-derived inoculation could represent not only a soil-health intervention, but a broader strategy for climate adaptation and mitigation in vulnerable dryland regions.

Together, these four questions define the tests needed to determine whether biocrust microbiomes can function as durable, ecosystem-level interventions in dryland agriculture. They also share a dimension of scale: the appropriate intervention and the outcomes it can realistically deliver, will differ between a small, intensively managed market garden and an extensive monoculture, and landscape-level benefits such as dust suppression and climate mitigation may require deployment well beyond a single field. Answering them will require tighter integration among microbial ecology, restoration ecology, and agronomy, particularly in climate-stressed arid regions where water scarcity, soil degradation, and food insecurity are intensifying. More broadly, these tests will determine whether dryland bioinoculation should continue to be designed around transferable traits in isolated strains, or instead around naturally assembled microbial ecosystems shaped by selection for persistence and multifunctionality under arid stress. This extends an emerging principle in microbiome engineering, that selection can be applied to whole communities rather than individual strains [20], by treating the agroecosystem itself as the selective filter. In that sense, biocrusts offer not only a candidate inoculant source for drylands, but also a model system for testing whether ecological organization is the relevant design unit for durable microbial interventions more generally. If this proves true, they may provide a conceptual foundation for circular, regenerative agriculture that supports food security in drylands worldwide.

## Data Availability

The authors declare that no data were generated or analyzed for this article.
